# A Minimal Model Describing Hexapedal Interlimb Coordination: The Tegotae-Based Approach

**DOI:** 10.3389/fnbot.2017.00029

**Published:** 2017-06-09

**Authors:** Dai Owaki, Masashi Goda, Sakiko Miyazawa, Akio Ishiguro

**Affiliations:** ^1^Research Institute of Electrical Communication, Tohoku UniversitySendai, Japan; ^2^Japan Science and Technology Agency, CRESTSaitama, Japan

**Keywords:** hexapedal locomotion, interlimb coordination, local sensory feedback, central pattern generator (CPG), Tegotae

## Abstract

Insects exhibit adaptive and versatile locomotion despite their minimal neural computing. Such locomotor patterns are generated via coordination between leg movements, i.e., an interlimb coordination, which is largely controlled in a distributed manner by neural circuits located in thoracic ganglia. However, the mechanism responsible for the interlimb coordination still remains elusive. Understanding this mechanism will help us to elucidate the fundamental control principle of animals' agile locomotion and to realize robots with legs that are truly adaptive and could not be developed solely by conventional control theories. This study aims at providing a “minimal" model of the interlimb coordination mechanism underlying hexapedal locomotion, in the hope that a single control principle could satisfactorily reproduce various aspects of insect locomotion. To this end, we introduce a novel concept we named “Tegotae,” a Japanese concept describing the extent to which a perceived reaction matches an expectation. By using the Tegotae-based approach, we show that a surprisingly systematic design of local sensory feedback mechanisms essential for the interlimb coordination can be realized. We also use a hexapod robot we developed to show that our mathematical model of the interlimb coordination mechanism satisfactorily reproduces various insects' gait patterns.

## 1. Introduction

Insects exhibit tremendously versatile gait patterns owing to their locomotion speed and physical/environmental conditions (Hughes, 1957; Graham, 1972, 1977; Cruse, 1976; Foth and Graham, 1983a,b; Dean, 1991; Zollikofer, 1994a,b,c; Noah et al., 2004; Goldman et al., 2006; Sponberg and Full, 2008; Grabowska et al., 2012; Wosnitza et al., 2013). These locomotor patterns are generated via their interlimb coordination mechanism. Biological findings suggest that interlimb coordination in hexapedal locomotion is controlled largely in a decentralized manner by neural networks located in thoracic ganglia (Pearson and Iles, 1969, 1973; Bässler and Wegner, 1983; Dean, 1989; Brekowitz and Laurent, 1996). Thus, clarifying this interlimb coordination mechanism is expected to allow us to obtain the key to understanding the control principle underlying animals' agile locomotion and for realizing truly adaptive legged robots that could not be realized solely by conventional control methods.

Aiming to elucidate the mechanism responsible for the interlimb coordination in hexapedal locomotion, various studies have been conducted to date by focusing on specific insects, e.g., stick insects (Graham, 1972, 1977; Cruse, 1976; Foth and Graham, 1983a,b; Dean, 1991; Grabowska et al., 2012) and cockroaches (Hughes, 1957; Pearson and Iles, 1969; Noah et al., 2004; Goldman et al., 2006; Sponberg and Full, 2008) and/or by focusing on control paradigms, e.g., *central pattern generators* (CPGs) (Pearson and Iles, 1973; Bässler and Wegner, 1983; Bässler, 1986, 1993; Ryckebusch and Laurent, 1993; Büschges et al., 1995, 2004; Bässler and Büschges, 1998; Büschges, 2005; Borgmann et al., 2009; Daun-Gruhn and Büschges, 2011; Marder and Bucher, 2011) and *chains of reflexes* (Cruse, 1983, 1990; Cruse et al., 1998; Dürr et al., 2004; Schilling et al., 2013). The knowledge obtained from these past studies deepened biological understanding of the interlimb coordination mechanism greatly; however, the diversity of these approaches may have confused roboticists who want to build adaptive insect-like hexapod robots via bio-inspired approaches (Kimura et al., 1993; Beer et al., 1997; Altendorfer et al., 2001; Ritzmann et al., 2004; Ambe et al., 2013; Manoonpong et al., 2013).

In order to address this problem, in this study, we attempt to capture the control principle essential to understanding the interlimb coordination in a concise form that could help bridge the gap between biologists and roboticists, in the hope that a single control principle could adequately reproduce various aspects of insect locomotion. Since reduction is required for understanding the essence, we build a “minimal model” of the interlimb coordination mechanism on the basis of a mathematically tractable highly abstract model. To this end, we employ a unique approach in this study. We introduce a novel concept we named “Tegotae,” a Japanese concept describing the extent to which a perceived reaction matches an expectation. We then introduce a Tegotae function, which is a function that quantitatively measures Tegotae, whereby we can design a decentralized interlimb coordination mechanism in a systematic manner. We validated the Tegotae-based interlimb coordination model by using a physical hexapod robot that we developed. We confirmed that the model adequately reproduced various aspects of insect locomotion patterns. We expect that our minimal model, systematically derived from the concept of Tegotae, will provide substantial insight into the essence of the interlimb coordination mechanism to roboticists as well as biologists.

The following section presents the materials and methods used in this study. First, we describe a basic building block for the interlimb coordination mechanism. Second, we explain the Tegotae concept and the design scheme of local sensory feedback using the Tegotae-based approach. Third, we explain the developed robotic platform in detail. Section 3 presents the experimental results to validate our Tegotae-based control for the interlimb coordination mechanism. Finally, in Section 4, we discuss our results and future work.

## 2. Materials and methods

### 2.1. Basic building block of interlimb coordination mechanism employed

To capture the control principle essential for the interlimb coordination mechanism, which works largely in a decentralized manner in insects' thoracic ganglia, it is important to determine a basic building block to be used for the distributed control system. From a control perspective, past studies have intensively argued mainly from the viewpoint of two distinct control paradigms: chains of reflexes (Cruse, 1983, 1990; Cruse et al., 1998; Dürr et al., 2004; Schilling et al., 2013) and CPGs (Pearson and Iles, 1973; Bässler and Wegner, 1983; Bässler, 1986, 1993; Ryckebusch and Laurent, 1993; Büschges et al., 1995, 2004; Bässler and Büschges, 1998; Büschges, 2005; Borgmann et al., 2009; Daun-Gruhn and Büschges, 2011; Marder and Bucher, 2011). In the chain-of-reflex approach, a control system is modeled by using many chained discontinuous reflexive events, in which locomotion can be generated purely from the interaction between sensory feedback signals and the body. However, the discontinuity in this approach may impede mathematical tractability (Daun-Gruhn and Büschges, 2011). In contrast, in the CPG approach, a control system is modeled by using directly coupled oscillators to generate feedforward motor commands, based on a continuous dynamical system, i.e., a set of differential equations, for the interlimb coordination. Considering the mathematical tractability stemming from a continuous model, we employ the CPG approach as a control paradigm. The CPG approach offers various ways to model a basic building block at different levels of abstraction (Ijspeert, 2008), ranging from detailed models using a single cell (Hodgkin and Huxley, 1952; Hellgren et al., 1992) to abstract oscillator models (Fitz-Hugh, 1969; Van der Pol, 1972; Kuramoto, 1984). Here we use a *phase oscillator* (Kuramoto, 1984) for each leg to build a minimal model of the interlimb coordination mechanism on the basis of a highly abstract model.

**Figure 1:**
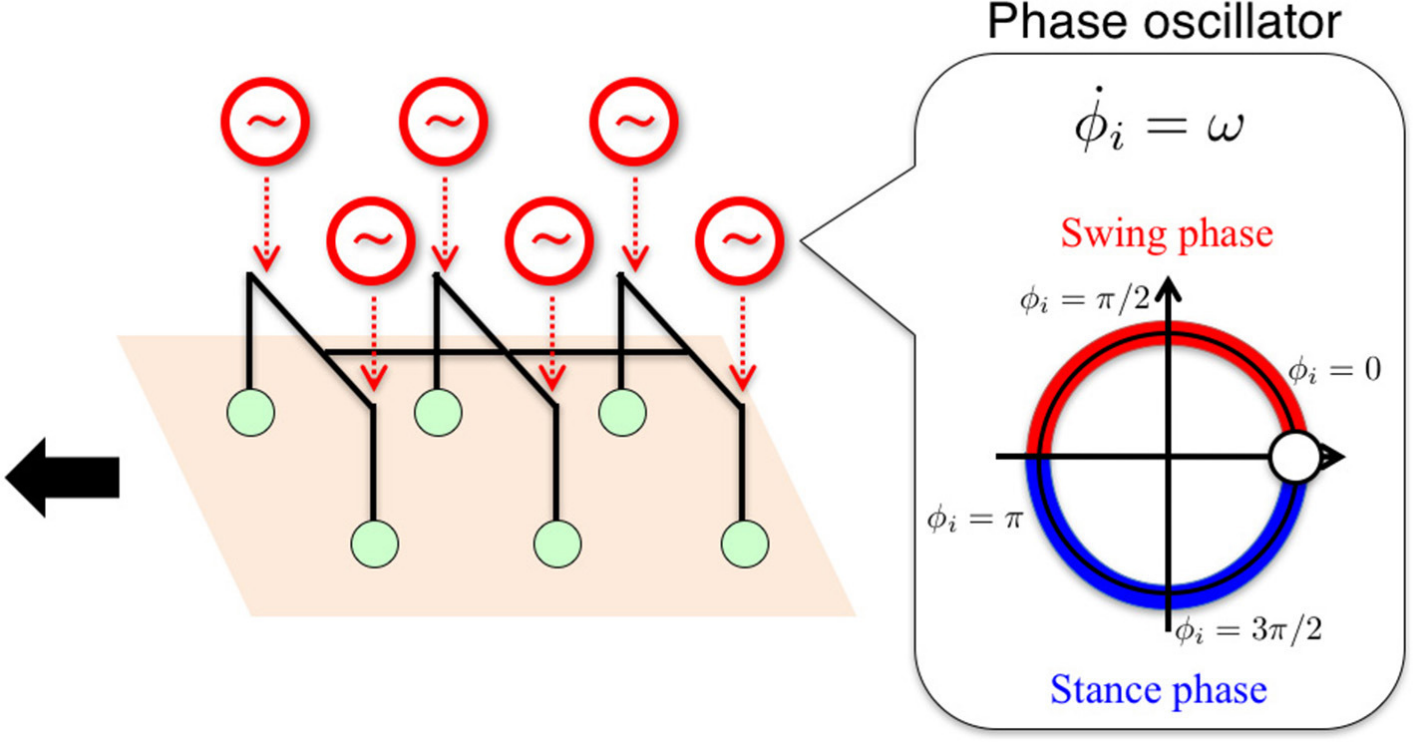
Schematic of the basic building block for the control system. We used a *phase oscillator* (Kuramoto, 1984) with local sensory feedback for each leg for hexapedal interlimb coordination. The *i*th leg is actively controlled (see Figure 5) according to ϕ_*i*_ such that the *i*th leg is in the swing phase when 0 ≤ ϕ_*i*_ < π and in the stance phase when π ≤ ϕ_*i*_ < 2π.

The time evolution of the oscillator phase is described by a differential equation as follows:

(1) $${\dot{\phi }}_{i}=\omega +f_{i},$$

where ω is the intrinsic angular velocity; ϕ_*i*_ is the phase of the oscillator implemented into the *i*th leg; and *f*_*i*_ is a local sensory feedback term, which plays an essential role in the interlimb coordination. This equation is one of the abstract oscillator models, i.e., the *Kuramoto model* (Kuramoto, 1984) (a case without coupling between oscillators and with local sensory feedback *f*_*i*_), which describes a one-dimensional, reduction model of oscillatory behaviors. Using the trigonometric functions (sin ϕ_*i*_, cos ϕ_*i*_, etc.) of oscillator phases enables us to generate a periodic motor command to control the legs of a robot. As an example of implementation, we describe the target angles ${\tilde{\theta }}_{yaw,i}$ and ${\tilde{\theta }}_{roll,i}$ for the proportional and derivative (PD) control of the motors (as explained in Section 2.4 and **Figure 6** in detail) through the following equations:

(2) $${\tilde{\theta }}_{yaw,i}\;=\;-A\;\cos\phi_{i},$$

(3) $${\tilde{\theta }}_{roll,i}=\;\left\{ \begin{array}{l} B\text{ sin }\phi_{i},\text{when}\;0\leq \phi_{i}<\pi ,\\ B\prime \text{ sin }\phi_{i},\text{when}\;\pi \leq \phi_{i}<2\pi , \end{array}\right.$$

where *A*, *B*, and *B*′ are user-defined parameters, describing amplitudes in the yaw and roll direction for leg motion (see Section 2.4 and Table 1). Thus, the *i*th leg is actively controlled according to ϕ_*i*_ such that the *i*th leg is in the swing phase when 0 ≤ ϕ_*i*_ < π, i.e., sin ϕ_*i*_ > 0, and in the stance phase when π ≤ ϕ_*i*_ < 2π, i.e., sin ϕ_*i*_ < 0, as shown in Figure 1. Below, we explain how we design local sensory feedback *f*_*i*_ by introducing the concept of “Tegotae” in a systematic manner.

**Table 1:** Parameters for each experiment.

Common parameters	Gain for *T*_1_	σ_1_	0.2
	Weight for anterior $N_{j}^{V}$	*k*_*a*_	0.7
	Weight for posterior $N_{j}^{V}$	*k*_*p*_	0.1
	Weight for contralateral $N_{j}^{V}$	*k*_*c*_	0.2
	Leg amplitude of anterior-posterior swing motion	*A*	15°
	Leg amplitude of up-down swing motion	*B*	20°
	Leg amplitude of stance motion	*B*′	5°
Section 3.1	Intrinsic angular velocity	ω	2.0
	Gain for *T*_2_	σ_2_	1.2
Section 3.2	Intrinsic angular velocity	ω	2.0 → 4.0 (40.0–42.0 s)
	Gain for *T*_2_	σ_2_	1.2
Section 3.3	Intrinsic angular velocity	ω	2.0 → 4.0 (40.0–42.0 s)
	Gain for *T*_2_	σ_2_	1.2
	Load		500 g
Section 3.4	Intrinsic angular velocity	ω	2.0
	Gain for *T*_2_	σ_2_	1.2
Section 3.5	Intrinsic angular velocity	ω	2.0
	Gain for *T*_2_	σ_2_	0.0

### 2.2. Tegotae and tegotae function

Here we explain the core concept Tegotae in detail. Tegotae is a novel concept describing the extent to which a perceived reaction matches an expectation (intention) of a controller. For ease of understanding, let us explain it metaphorically. Imagine you want to lean against a wall nearby. Note that what you want to do, i.e., leaning against the wall, is regarded as the intention of the controller, i.e., your nervous system. When you lean against the wall, if you feel that the reaction force from the wall is sufficient for supporting your body, we say “good” Tegotae is obtained. If the reaction force you receive is insufficient (imagine the wall were a curtain/screen for example), “bad” Tegotae is obtained. Notice that Tegotae stems not only from the reaction received from the environment, but also from the consistency between the perceived reaction and the intention/expectation of the controller, i.e., what the controller wants to do.

Now the question is how to quantify Tegotae. Of course, there are various ways to accomplish this. As the initial step of the investigation, we quantify Tegotae in the simplest mathematical form, i.e., a function based on the type of separation of variables as follows:

(4) $$T_{i}(\phi_{i},N)=C(\phi_{i})S(N).$$

Hereafter, we refer to the function *T*_*i*_ as the “Tegotae function”—a function that quantitatively measures Tegotae. ϕ_*i*_ is a control variable (in this case the phase of the oscillator), and *N* is the sensory information obtained from multiple sensors embedded in the body. Note that, the Tegotae function *T*_*i*_ is expressed as the product of two functions *C*(ϕ_*i*_) and *S*(*N*): the former is a function expressing the intention of the controller, and the latter denotes the reaction obtained from the environment. Here, we design *T*_*i*_ such that it becomes more positive when enhanced Tegotae is detected. Next, we explain how we can design the sensory feedback term *f*_*i*_ by using *T*_*i*_.

### 2.3. Tegotae-based control

Given that the Tegotae function is defined, the local sensory feedback term *f*_*i*_ is designed in such a way that the control system modulates ϕ_*i*_ in order to increase the amount of Tegotae received. Thus, because a continuous system is used, *f*_*i*_ is expressed simply as the partial derivative of the Tegotae function *T*_*i*_ with respect to the control variable ϕ_*i*_, as follows:

(5) $$f_{i}=\frac{\partial T_{i}(\phi_{i},N)}{\partial \phi_{i}}.$$

Note that we can systematically design decentralized controllers by only designing the Tegotae functions required.

Now, the question is how to define *T*_*i*_(ϕ_*i*_, *N*) to satisfactorily reproduce the hexapedal interlimb coordination observed in insect locomotion. In this study, we define *T*_*i*_(ϕ_*i*_, *N*) as follows:

(6) $$T_{i}(\phi_{i},N)=\sigma_{1}T_{i,1}(\phi_{i},N)+\sigma_{2}T_{i,2}(\phi_{i},N),$$

(7) $$T_{i,1}(\phi_{i},N)=(-\text{sin }\phi_{i})N_{i}^{V},$$

(8) $$T_{i,2}(\phi_{i},N)=\text{sin }\phi_{i}\left(\frac{1}{n_{L}}\sum_{j\in L(i)}^{n_{L}}k_{j}N_{j}^{V}\right).$$

**Figure 2:**
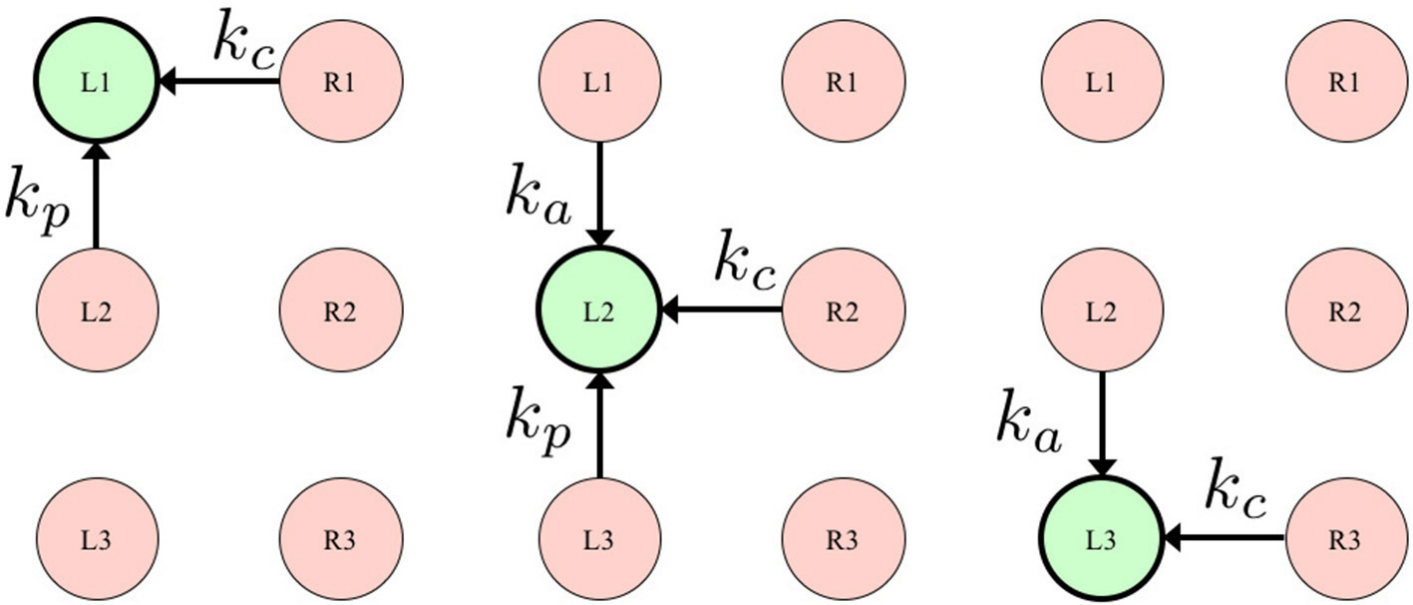
Definition of *L*(*i*), describing a set consisting of the legs neighboring the *i*th leg. The left, center, and right figures show the set of left fore (L1), left middle (L2), and left hind (L3) legs, respectively. *k*_*j*_ (*k*_*a*_, *k*_*p*_, *k*_*c*_ ≥ 0) denotes the weight for each GRF *N*_*j*_.

As Equation (6) indicates, *T*_*i*_(ϕ_*i*_, *N*) consists of two Tegotae functions, *T*_*i*, 1_(ϕ_*i*_, *N*) and *T*_*i*, 2_(ϕ_*i*_, *N*), both of which are linearly coupled via the positive constants σ_1_ and σ_2_. The suffix *i* denotes the leg number (*i*:1, 2, …, 6). Sensory information *N* consists of vertical ground reaction forces (GRFs) acting on each leg $N={\left[N_{1}^{V},N_{2}^{V},\ldots ,N_{6}^{V}\right]}^{T}$. *L*(*i*) denotes a set consisting of the legs neighboring the *i*th leg, and *n*_*L*_ is the number of elements in *L*(*i*) and *k*_*j*_ (*k*_*a*_, *k*_*p*_, *k*_*c*_ ≥ 0) denotes the weight for each GRF $N_{j}^{V}$, as shown in Figure 2. Further, we present a detailed explanation of the approach we followed when designing these two Tegotae functions.

**Figure 3:**
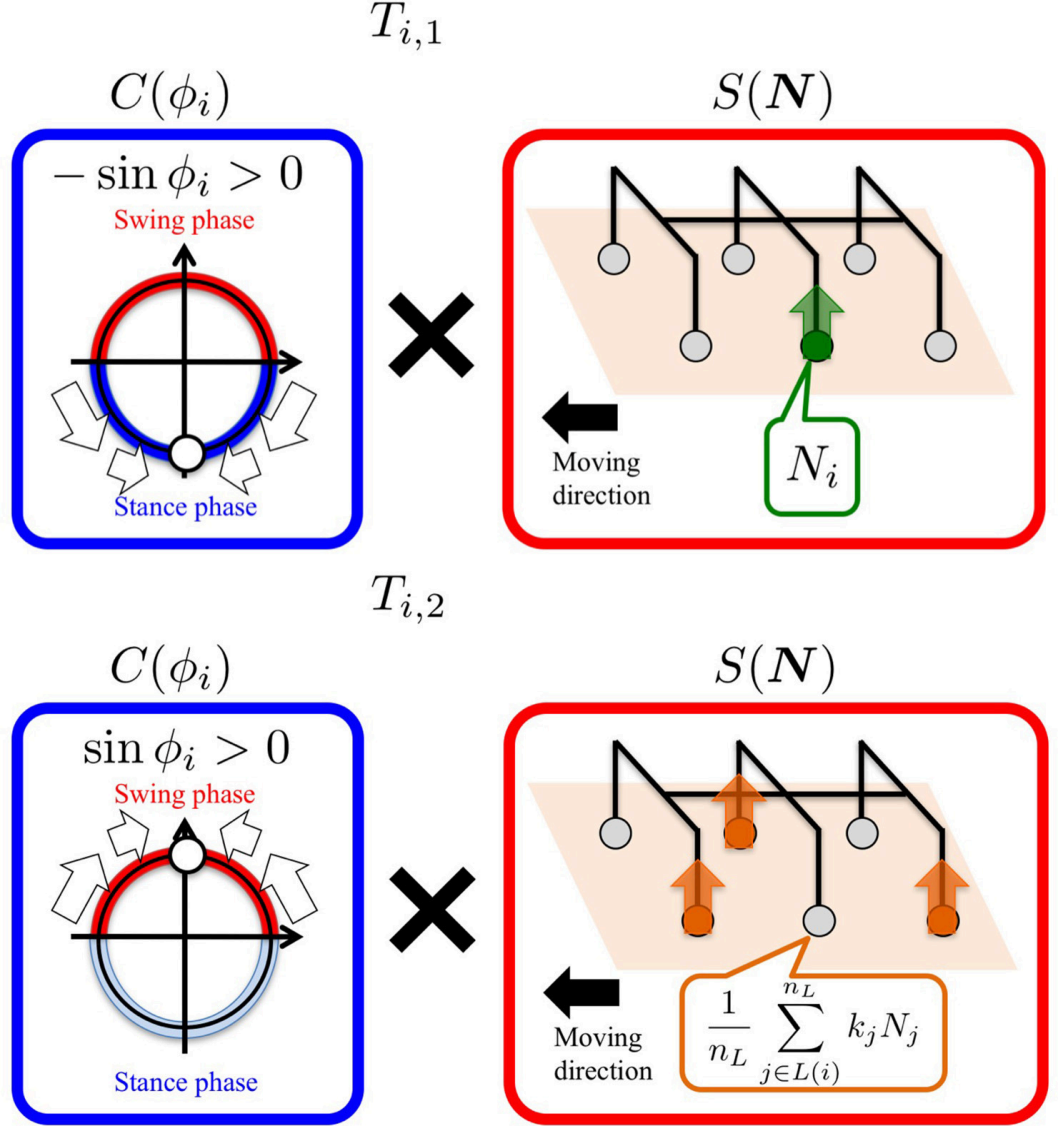
Definition of the “Tegotae” functions, which are expressed as the product of *C*(ϕ_*i*_) and *S*(*N*). We design *T*_*i*_ such that it becomes more positive when enhanced Tegotae is detected. The upper and lower figures show the *T*_*i*, 1_ and *T*_*i*, 2_ functions for the left middle leg (L2), respectively.

*T*_*i*, 1_ quantifies Tegotae on the basis of the information that is only locally available at the corresponding leg; when the local controller intends to be in the stance leg (−sin ϕ_*i*_ > 0), and results in receiving a ground reaction force ($N_{i}^{V}>0$) (Figure 3, top), *T*_*i*, 1_ evaluates this situation as “good” Tegotae, and returns a positive value.

On the other hand, *T*_*i*, 2_ quantifies Tegotae on the basis of the relationship between the movements of the corresponding leg and its neighboring legs; when the local controller intends to be in the swing phase (sin ϕ_*i*_ > 0) and its neighboring legs offer good support to the body at that time ($\frac{1}{n_{L}}\overset{n_{L}}{\underset{j\in L\left(i\right)}{\sum }}k_{j}N_{j}^{V}>0$) (Figure 3, bottom), *T*_*i*, 2_ evaluates that the corresponding leg adequately establishes a relationship with its neighboring legs and returns a positive value.

By substituting Equations (6–8) into Equations (1) and (5), we obtain our interlimb coordination mechanism as follows:

(9) $${\dot{\phi }}_{i}=\omega -\sigma_{1}N_{i}^{V}\text{cos }\phi_{i}+\sigma_{2}\left(\frac{1}{n_{L}}\sum_{j\in L(i)}^{n_{L}}k_{j}N_{j}^{V}\right)\text{cos }\phi_{i}.$$

Introduction of the Tegotae-based approach enables us to easily design a minimal model for hexapedal interlimb coordination in a systematic manner.

### 2.4. Robotic platform for the validation of proposed control scheme

**Figure 4:**
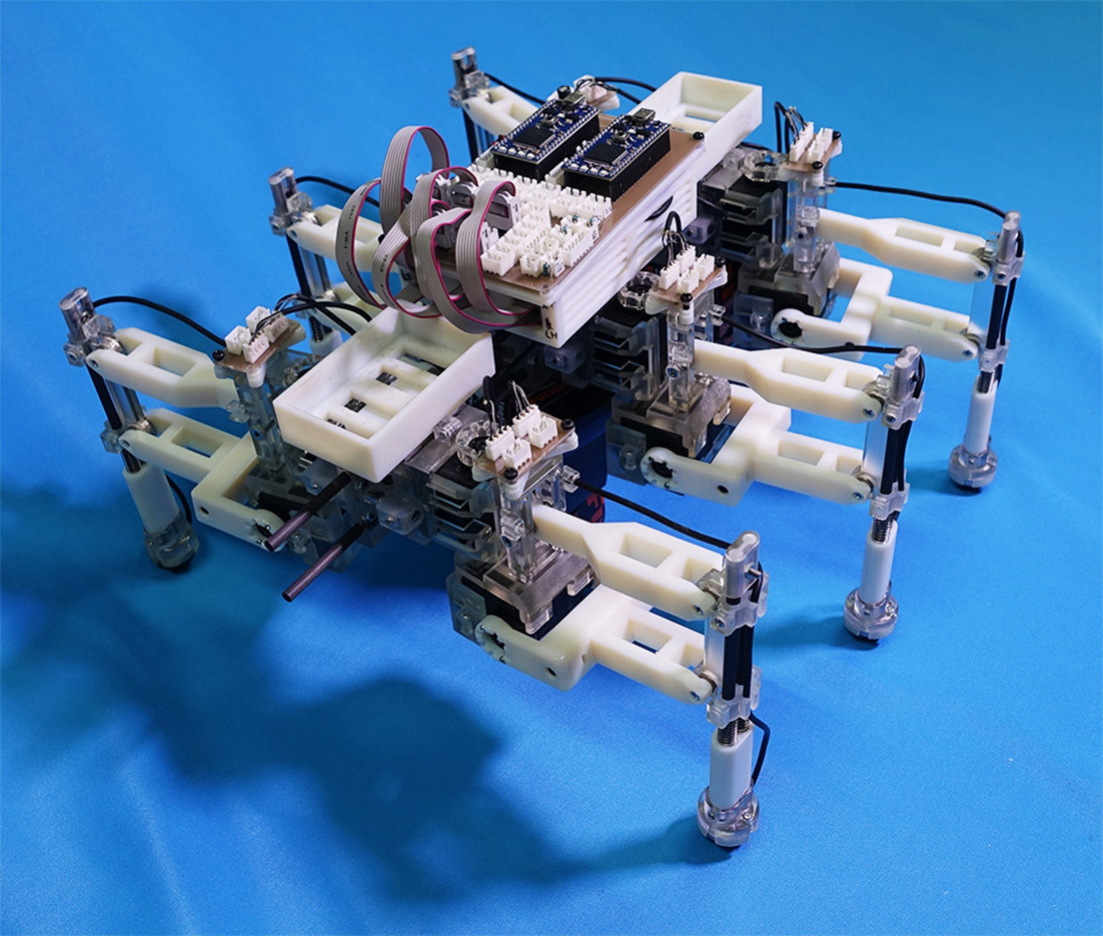
Hexapod robot developed for the study. The robot is 0.40 m long, 0.30 m wide, 0.20 m high, and weighs 2.4 kg.

**Figure 5:**
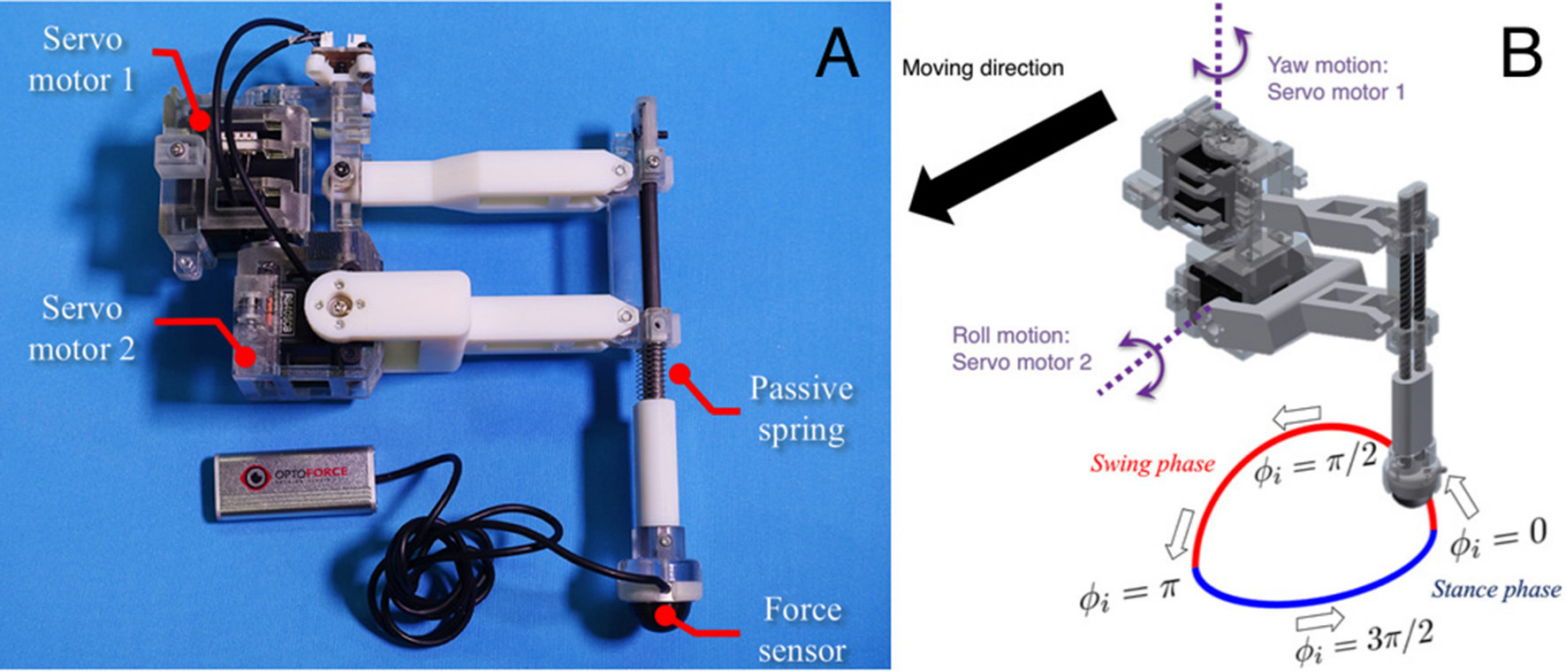
Detailed structure of the leg segment of the robot. **(A)** The leg consists of carbon fiber rods and ABS resin printed using a 3-D printer. The feet contain three-axis force sensors to detect GRFs. **(B)** Each leg is equipped with two servo motors, which generate leg motion during the swing and stance phases according to the corresponding oscillator phase.

**Figure 6:**
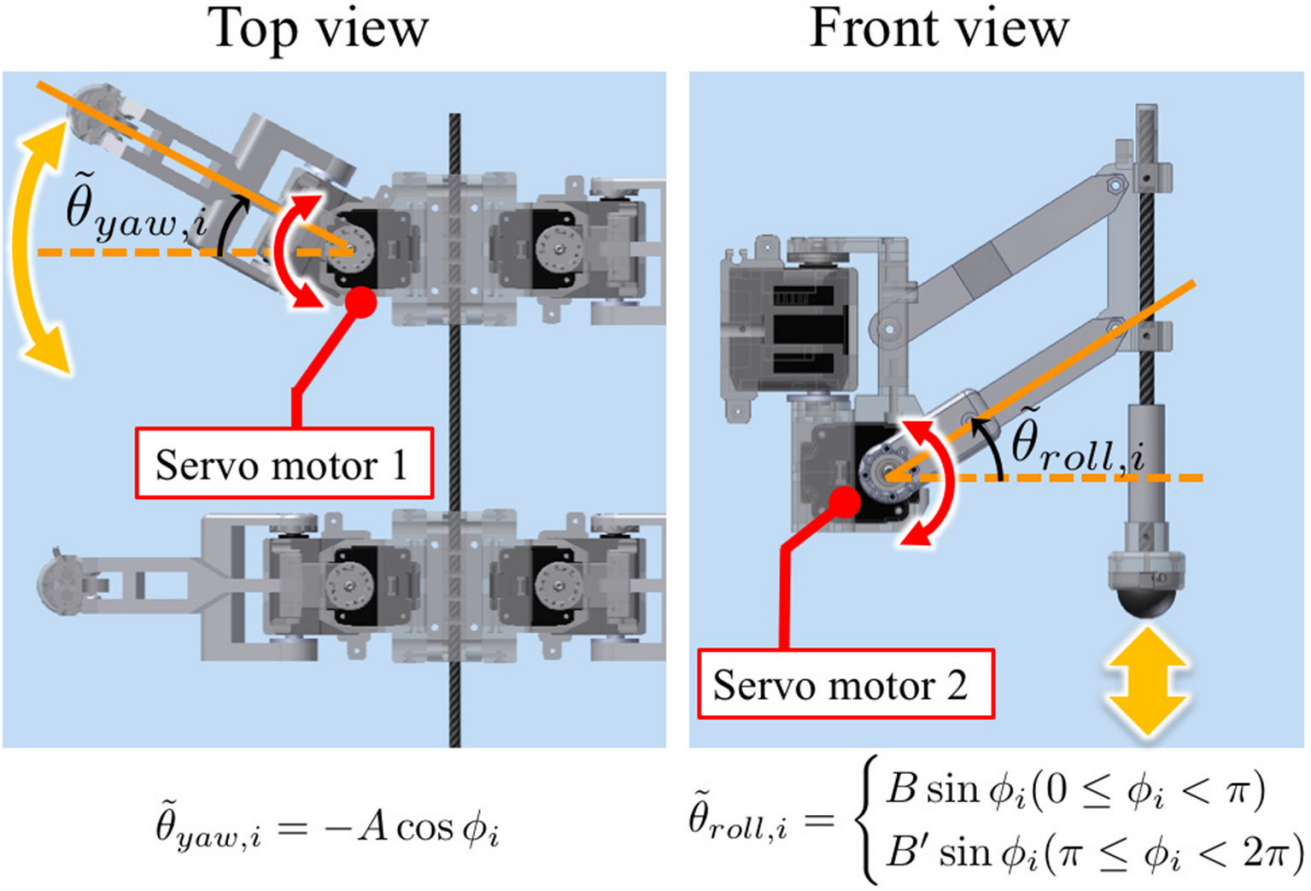
Leg trajectory for a single leg, where ${\tilde{\theta }}_{yaw,i}$ and ${\tilde{\theta }}_{roll,i}$ denote the target angles for proportional and derivative (PD) control of the motors in the yaw and roll directions, respectively. Based on this control scheme, we can generate periodic leg motion as shown in Figure 5B.

Figure 4 shows the structure of our hexapod robot. The robot consists of six leg segments (Figure 5) and a body segment. The robot is 0.40 m long, 0.30 m wide, 0.20 m high, and weighs 2.4 kg. The leg and body consist of carbon fiber rods and acrylonitrile butadiene styrene (ABS) resin printed using a 3-D printer. For each leg, we used two servo motors (Futaba Corporation, Japan: RS405CB), which generate leg motion during the swing and stance phases according to the corresponding oscillator phase (Figure 5B). As shown in Figure 6, we describe the target angles ${\tilde{\theta }}_{yaw,i}$ and ${\tilde{\theta }}_{roll,i}$ for proportional and derivative (PD) control of the motors through the following equations:

(10) $${\tilde{\theta }}_{yaw,i}\;=\;-A\text{ cos }\phi_{i},$$

(11) $${\tilde{\theta }}_{roll,i}\;=\;\left\{ \begin{array}{l} B\text{ sin }\phi_{i},\text{when}\;0\leq \phi_{i}<\pi ,\\ B\prime \text{ sin }\phi_{i},\text{when}\;\pi \leq \phi_{i}<2\pi . \end{array}\right.$$

Based on this control scheme, we can generate periodic leg motion as shown in Figure 5B. From the viewpoint of neurophysiological findings for a locomotor CPG system in animals (Lafreniere-Roula and McCrea, 2005; Rybak et al., 2016), Equation (9) corresponds to the rhythm generator (RG) and Equations (10) and (11) correspond to a pattern formation (PF) network in the two-level CPG concept. For the robot, we choose parameter values *A, B, B*′ for the geometric path of the foot by tuning them through trial and error as shown in Table 1. We employ passive springs (MISUMI Corporation: WM8-20, 2.9 N/mm) in each leg for shock absorption. Furthermore, we use three-axis force sensors (OptoForce Ltd., Hungary: OMD-20-SE-40N) in the feet of the robot to detect ground reaction forces (GRFs), as shown in Figure 5A.

The body contains a main control board. We calculate the oscillator phase in each leg by using microcontrollers (mbed NXP LPC1768) on the main control board. We manipulated each servo motor installed in the legs using proportional-derivative (PD) control as explained above.

## 3. Experimental results

To verify the proposed control scheme in the real world, we conducted five experiments: (i) steady walking, (ii) gait transition according to locomotion speed, (iii) adaptability to change in weight distribution, (iv) adaptability to leg amputation, and (v) effect of local sensory feedback. The control parameters that were used in experiments with the hexapod robot (Sections 3.1–3.5) are listed in Table 1. We conducted over 10 trials for each experiment: each trial was conducted on a treadmill for a period of 50 s using randomly selected initial phases.

### 3.1. Steady walking

**Figure 7:**
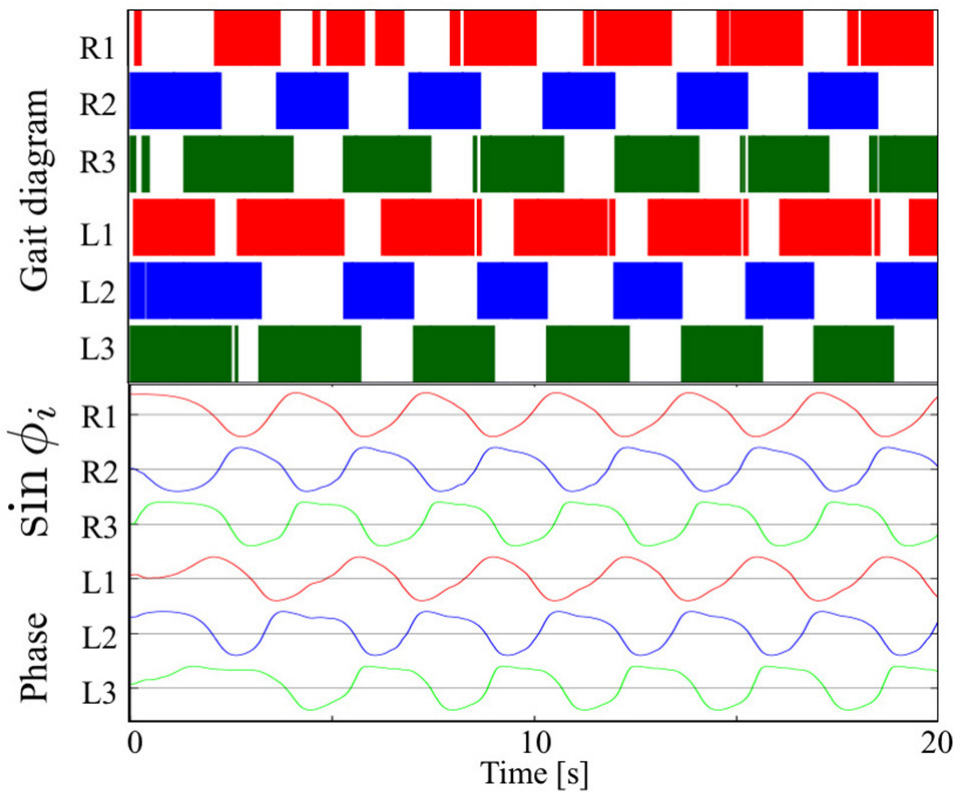
Upper graph: Gait diagram. Lower graph: Corresponding phase sin ϕ_*i*_. The gait pattern rapidly converges from the initial phase relationship to a tetrapod gait, in which the ipsilateral feet touch the ground in the order hind, middle, and fore legs, within approximately two periods. The results confirmed that the initial patterns converged to the same gait patterns from any initial phase relationship (in 10 out of 10 trials: 100%).

Figure 7 shows the results of measurements conducted when our robot was engaged in steady walking. Here, we set the parameter ω = 2.0 rad/s. Figure 7 shows the gait diagram (upper graph) and time evolution of the oscillator phases of the legs (lower graph, sin ϕ_*i*_) for the period 0.0–20.0 s. In the gait diagram, the colored regions represent the stance phase, which is distinguished by using the threshold data value (1.5 N: less than 10% of the maximum force detected) from the force sensor. Hereafter, we use the gait diagrams and movies (i.e., Movies S1–S3) recorded by a video camera as a qualitative evaluation index and the average duty factors (the ratio of the stance phase to one period) as a quantitative evaluation index. For the quantitative analysis, the duty factors obtained by the gait diagrams reflect the direction of the robot motion (i.e., straightness) because the asymmetric duty factors in the left and right legs indicate turning in the locomotion. Moreover, the duty factors indirectly represent the foot point velocity during the locomotion because the leg trajectory of our robot is determined in response to oscillator phases (Figure 6). Thus, the data of the duty factors from the gait diagrams indirectly include physical information about the speed and the direction of the locomotion (see SM for more details). The gait pattern rapidly converges from the initial phase relationship to a tetrapod gait—the ipsilateral feet touch the ground in the order of hind, middle, and fore legs—within approximately two periods. Furthermore, we tested the effect of the variation in the initial oscillator phases on the gait patterns. The results confirmed that the initial patterns converged to the same gait patterns from any initial phase relationship (in 10 out of 10 trials: 100%).

### 3.2. Gait transitions according to locomotion speed

**Figure 8:**
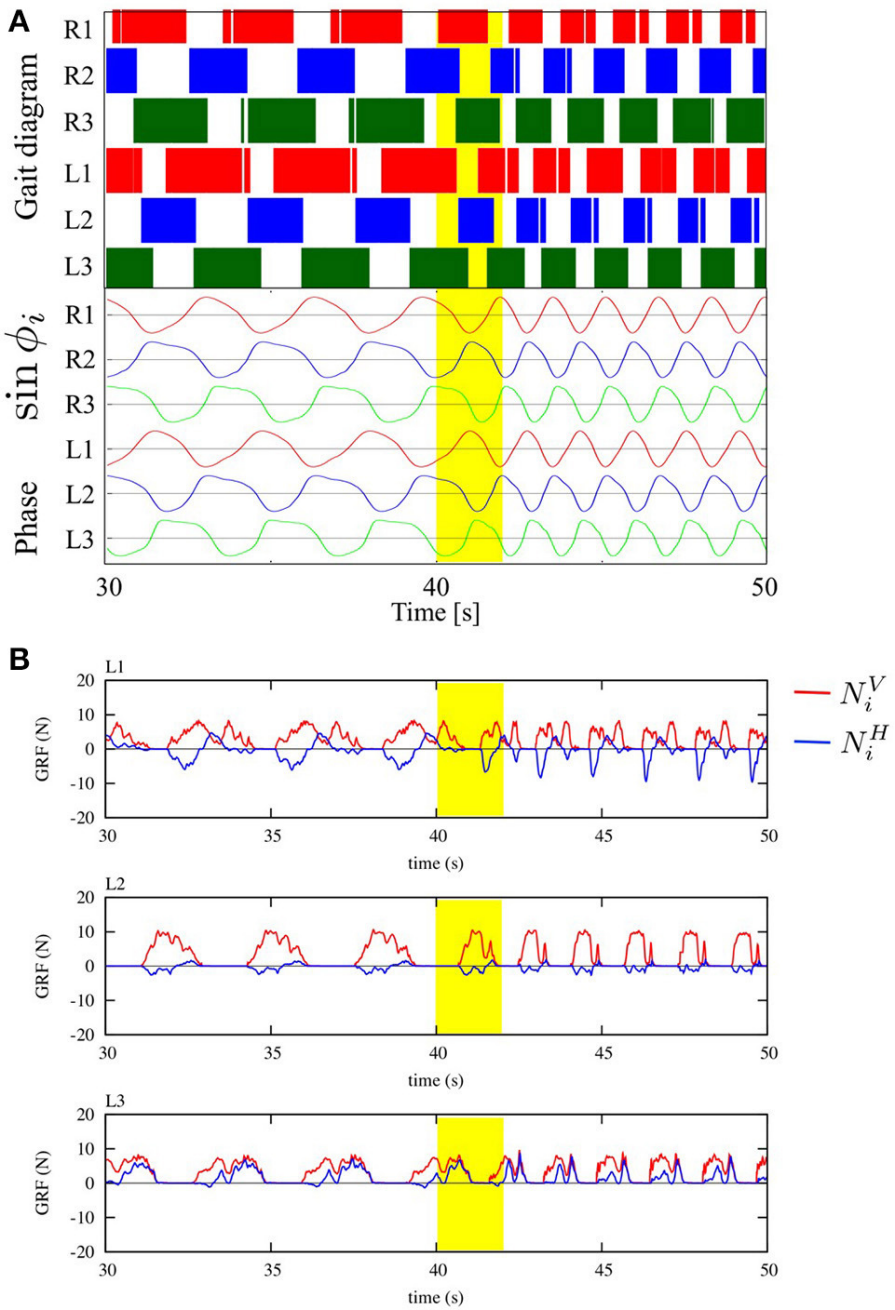
**(A)** Upper graph: Gait diagram. Lower graph: Corresponding phase sin ϕ_*i*_. We found spontaneous transition from the gait of a tetrapod to that of a tripod, in which the (L1, R2, L3) and (R1, L2, R3) feet alternately touch the ground in anti-phase, by changing only parameter ω from 2.0 to 4.0 rad/s in the period from 40.0 to 42.0 s (yellow highlight in the graph, Movie S1). We confirmed the same result for the gait transition in all 10 trials (10/10: 100%). **(B)** The profile of vertical and horizontal GRFs ($N_{i}^{V}$ and $N_{i}^{H}$). The upper, middle, and lower graphs show the GRF profile of the front (L1), middle (L2), and hind (L3) legs, respectively.

We tested the ability of the proposed control scheme to change the gait patterns according to the locomotion speed by linearly changing the parameter ω from 2.0 to 4.0 rad/s during the time period 40.0 to 42.0 s. Figure 8A shows the gait diagram (upper graph) and the time evolution of oscillator phases of legs (lower graph, sin ϕ_*i*_), during the time period 30.0–50.0 s in this experiment. After ω was chenged, the gait pattern spontaneously changed from that of a tetrapod to that of a tripod—the (L1, R2, L3) and (R1, L2, R3) feet alternately touch the ground in the anti-phase (Movie S1). Figure 8B shows the profile of vertical and horizontal GRFs ($N_{i}^{V}$ and $N_{i}^{H}$) in the same experiment. In this figure, the upper, middle, and lower graphs show the GRF profile of the front (L1), middle (L2), and hind (L3) legs, respectively. Furthermore, we confirmed this result for the gait transition in all 10 trials (10/10: 100%). The results indicate that leg coordination is appropriately modified according to the locomotion speed via Tegotae-based control.

### 3.3. Adaptability to change in weight distribution

**Figure 9:**
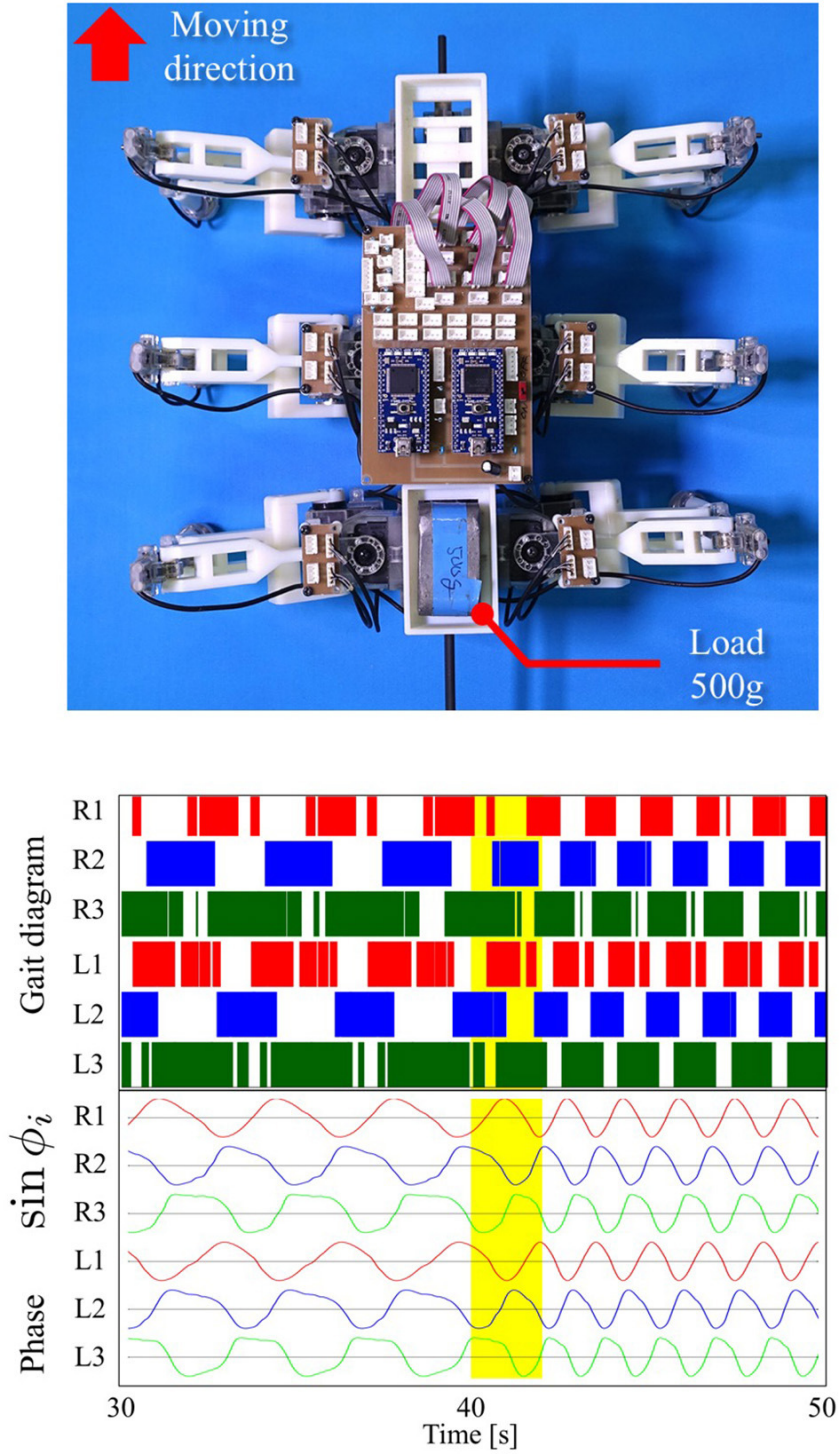
**Top:** Location of a load (500 g) applied to our robot. **Bottom:** Gait diagram and corresponding phase sin ϕ_*i*_. After changing ω, the gait pattern did not change to that of a tripod; instead, a tetrapod gait was maintained (Movie S2). We found the same results in 10 out of 10 trials (100%).

**Figure 10:**
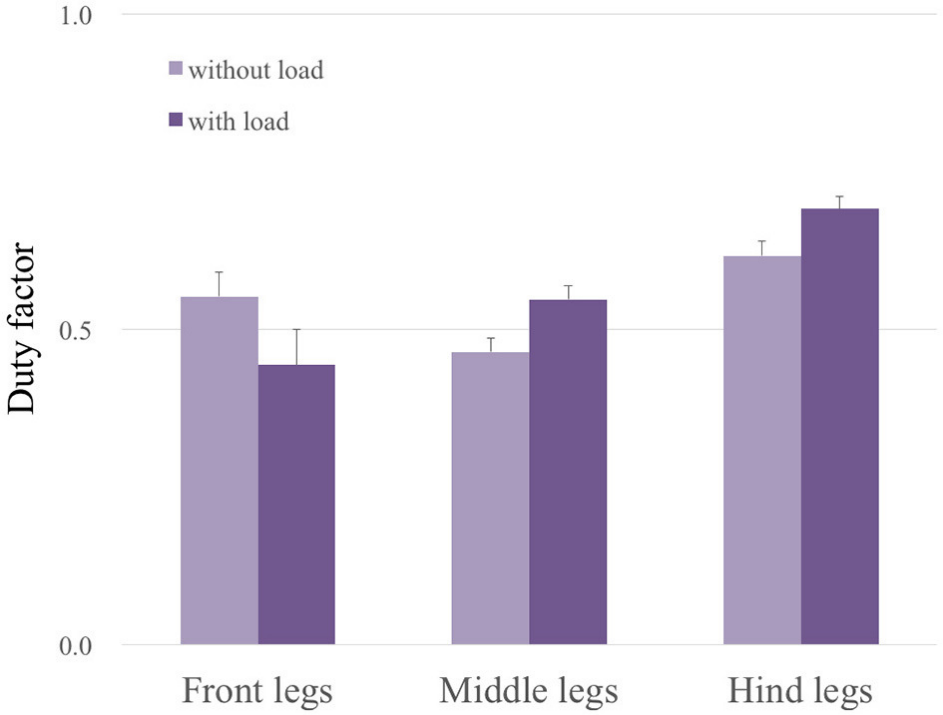
Average duty factor of each leg without and with a load through 10 trials (ω = 4.0 rad/s). This result indicates that the duty factors of the loaded hind legs and middle legs are larger than those of legs without a load, whereas the duty factor of the front legs becomes smaller.

Here, we show the adaptability of our robot to changes in weight distribution by applying a load (500 g) to the hind portion of the body (upper photograph in Figure 9). The lower graphs in Figure 9 show the experimental result. Here, we changed the parameter ω from 2.0 to 4.0 rad/s during the period 40.0 to 42.0 s as in the previous gait transition experiments (Section 3.2). After changing ω, the gait pattern did not change to that of a tripod; instead, a tetrapod gait was maintained (Movie S2). We obtained the same results in 10 out of 10 trials (100%). Figure 10 compares the average duty factor of the front, middle, and hind legs without and with the load for 10 trials (ω = 4.0 rad/s). The duty factor, which is the ratio of the stance phase to one period, was calculated by using the gait patterns during six periods for each trial. This result indicates that the duty factor of the loaded hind legs is larger than that of legs that do not bear any load. This result demonstrates the adaptability of our proposed control scheme to changes in the weight distribution without requiring prior data about these changes.

### 3.4. Adaptability to leg amputation

**Figure 11:**
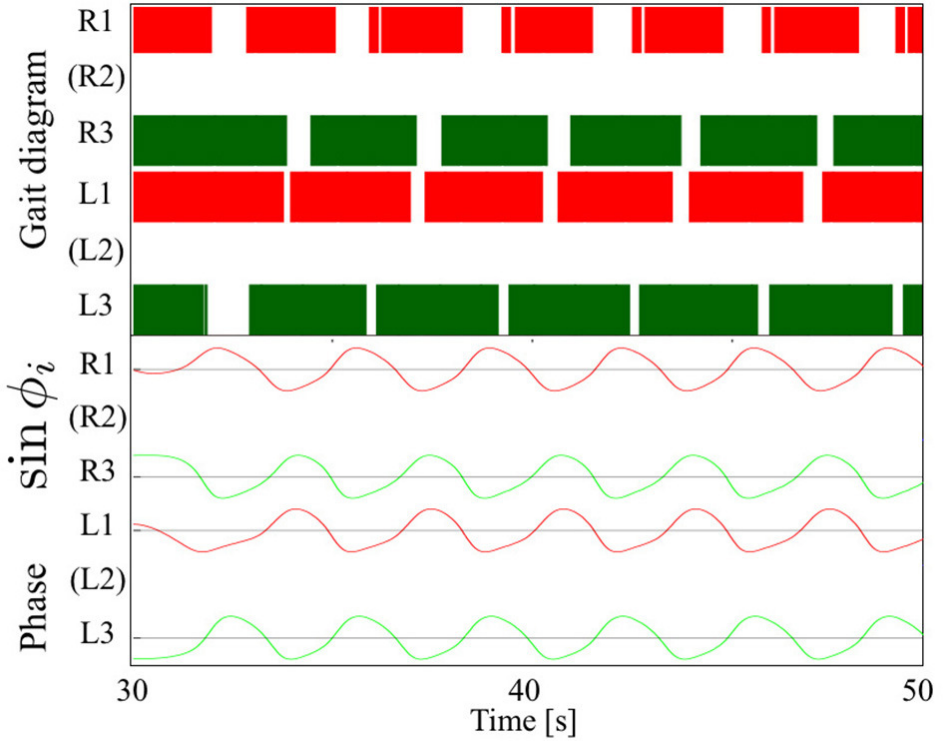
Upper graph: Gait diagram. Lower graph: Corresponding phase sin ϕ_*i*_. The gait patterns converged to a *trot* or an *L-S walk* gait (in 10 out of 10 trials: 100%) observed in quadrupeds, in which case the (L1, R3) and (R1, L3) feet alternately touch the ground in anti-phase (Movie S3).

**Figure 12:**
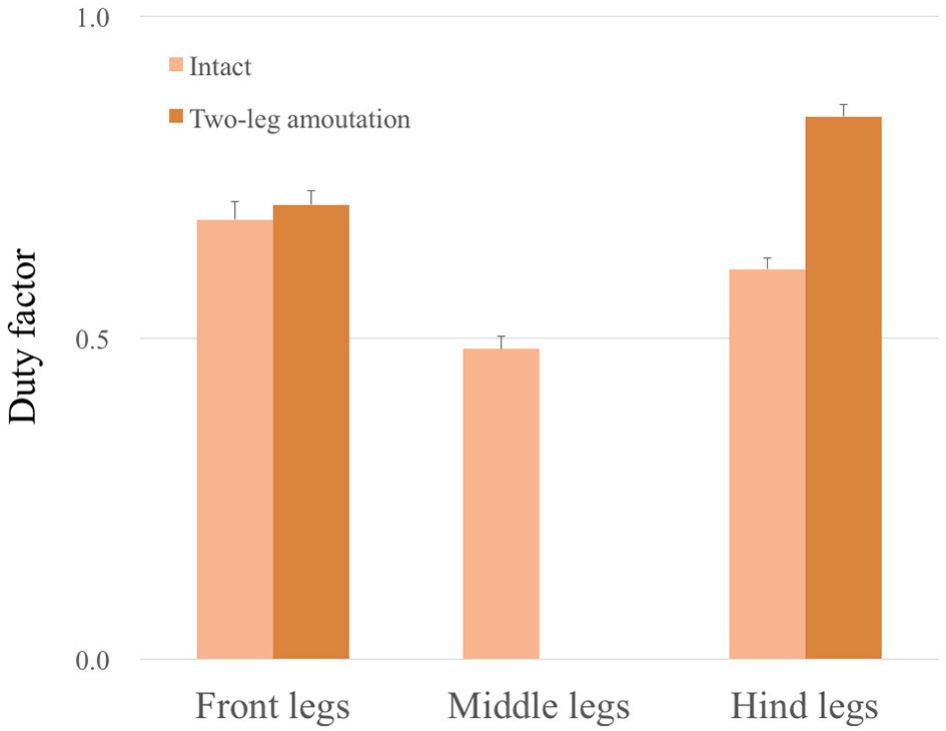
Average duty factor of front, middle, and hind legs in the leg amputation experiment in 10 trials. The duty factor of the hind legs mainly increased in the case of two-leg amputation experiments.

Figure 11 shows the experimental results of the leg amputation test after both of the middle legs were amputated. In spite of the amputation, the robot was able to continue walking. Furthermore, the gait patterns converged to a *trot* or an *L-S walk* gait observed in quadrupeds—*i. e*. the (L1, R3) and (R1, L3) feet alternately touch the ground in nearly anti-phase, or more precisely, focusing on the timing of touch down, the feet touch the ground in the order from L1, R3, R1, L3 (Movie S3). Figure 12 compares the average duty factor of the front, middle, and hind legs for 10 trials of the leg amputation experiment. The duty factor of each leg was modulated according to the remaining number of legs, which mainly resulted in increasing the duty factor of the hind legs. Furthermore, we confirmed that the initial patterns converged to the same gait patterns from any initial phase relationship (in 10 out of 10 trials: 100%). These results also indicate that the proposed control scheme can achieve interlimb coordination according to the physical properties of the robot's body in a self-organizing manner, without any predefined gait patterns.

### 3.5. Effect of local sensory feedback concerning neighboring legs

**Figure 13:**
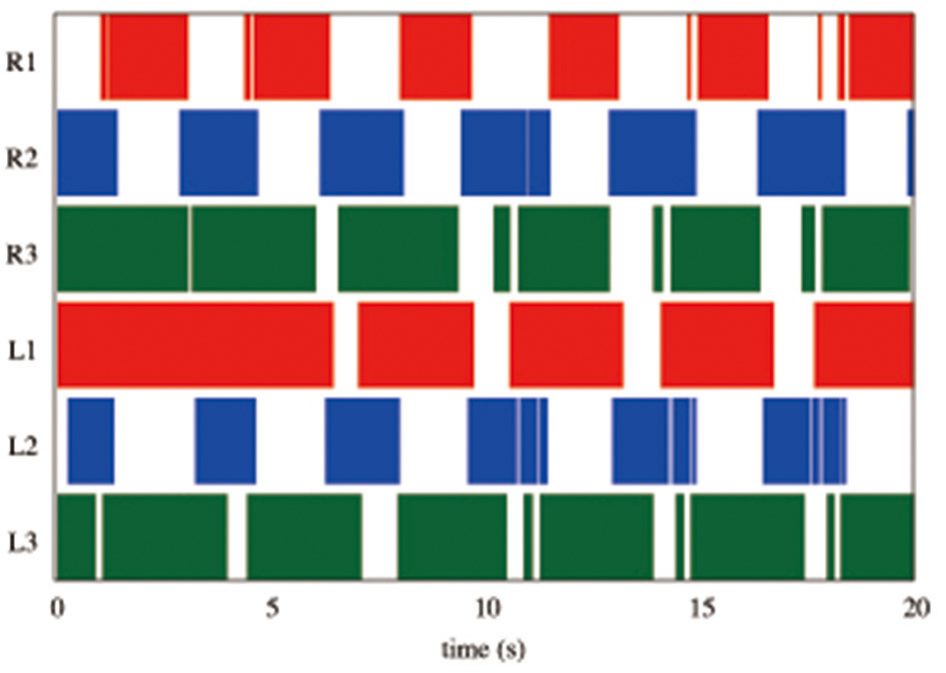
Experimental results: ω = 2.0, σ_1_ = 0.2, σ_2_ = 0. The gait patters mostly did not converge to insect-like gaits, e.g., tetrapod/tripod gaits, but converge to other patterns under many initial conditions (in 7 out of 10 trials: 70%). In these gaits, the left legs touched the ground in the order L3, L2, and L1 (hind to fore), whereas the right legs touched in the order R1, R2, and R3 (fore to hind).

The usefulness of our proposed local sensory feedback was verified based on the Tegotae approach by conducting experiments with the following conditions: we set the parameters ω = 2.0, σ_1_ = 0.2, σ_2_ = 0, which is a model similar to our previous model for quadrupeds (Owaki et al., 2012; Owaki and Ishiguro, 2017) or Barikhan's model for hexapod models (Barikhan et al., 2014). We conducted 10 trials in this experiment using randomly selected initial phases. Figure 13 shows the experimental results obtained using these parameters. The gait patterns mostly did not converge to insect-like gaits, e.g., tetrapod/tripod gaits, but converged to other patterns under many initial conditions (in 7 out of 10 trials: 70%) in this model. In these gaits, the left legs touched the ground in the order L3, L2, and L1 (hind to fore), whereas the right legs touched in the order R1, R2, and R3 (fore to hind). This result indicates that the model with only the second term in Equation (9) (similar to Barikhan's model) sometimes reproduced a gait pattern similar to that of insects, but its robustness against the initial conditions was insufficient.

## 4. Discussion

The purpose of this study was to provide a minimal model for the interlimb coordination in hexapedal locomotion based on a novel concept named Tegotae. Using the Tegotae-based approach has enabled us to show how we can design the local sensory feedback for a decentralized interlimb coordination mechanism in a systematic manner. Moreover, we have demonstrated that our hexapod robot, which was developed for the validation of the proposed control scheme, satisfactorily reproduced various aspects of insect locomotion, i.e., steady walking, gait transition according to locomotion speed, and adaptability to changes in weight distribution and to leg amputation. As shown in Figure 8B, the role arrangement of the fore, middle, and hind legs can be achieved via the interlimb coordination mechanism: (i) the fore legs mainly generate breaking forces ($N_{i}^{H}$ was mainly negative), (ii) middle legs mainly support the body ($N_{i}^{V}$ was larger than those for the other legs), and (iii) hind legs mainly generate propulsion forces ($N_{i}^{H}$ was mainly positive). Such adaptive behaviors are commonly observed for various species of insects, as shown in Table 2. This suggests that our Tegotae-based interlimb coordination model captures the essential mechanism for hexapedal interlimb coordination. As a control experiment, if we set the parameters σ_1_ = σ_2_ = 0, i.e., a condition without local sensory feedback, we can easily imagine that interlimb coordination did not occur, but the phase relationship between leg movement maintains the initial condition. Thus, in order to determine the usefulness of the proposed local sensory feedback, we verified the effect of the second and third terms of Equation (9) in Section 3.

**Table 2:** Observed adaptive behavior various species of insects have in common.

Velocity change	Our robot	Stick insect (Graham, 1972)	Fruit fly (Mendes et al., 2013)
	Tetrapod → tripod	Tetrapod → tripod	Tetrapod → tripod
Load on their body	Our robot	Cricket (results in the SM)	Fruit fly (Mendes et al., 2014)
	Tetrapod	Tetrapod	Tetrapod
Amputating two middle legs	Our robot	Cockroach (Hughes, 1957)	Stick insect (Graham, 1977; Grabowska et al., 2012)
	Trot/L-S walk	L-S walk	Wave/L-S walk

In the previous study on quadruped locomotion (Owaki et al., 2012; Owaki and Ishiguro, 2017), we have proposed a simple interlimb coordination rule that well reproduced various quadruped gait patterns and well explained the underlying mechanism. The second term in Equation (9) corresponds to the quadruped interlimb coordination rule. Inspired by our model, Barikhan et al. (2014) also implemented an almost identical mechanism for a hexapedal interlimb coordination model and verified its usefulness by reproducing some insect-like locomotion in simulations. However, although our experiments about the effect of the third term in Equation (9) in Section 3.5 indicate that the model with only the second term in Equation (9) sometimes reproduces a gait pattern similar to that of insects, but its robustness against the initial conditions was insufficient. This is because the local load information on quadrupeds is totally reflected by physical information throughout the whole body (Owaki et al., 2012; Owaki and Ishiguro, 2017), whereas that on hexapods does not sufficiently include physical information for interlimb coordination. Thus, we concluded that the third term in Equation (9), which used sensory information about load distribution in neighboring legs, is essential for the reproduction of insect-like gait patterns and gait transitions. Moreover, we have already reported the local sensory feedback mechanism in Equation (9), but we did not previously confirmed the gait transition from tetrapod to tripod and the adaptability to change in the weight distribution and leg amputation (Goda et al., 2016). Here, we newly introduce anterior-posterior asymmetry in the parameter *k*_*a*_ and *k*_*p*_, which mainly resulted in the stable gait transition according to locomotion speed, i.e., from tetrapod to tripod as well as the adaptability to change according to the weight distribution and as a results of leg amputation. Our main contribution is the versatility of reproduced behaviors concerning insects' locomotion: Barikhan's model (Barikhan et al., 2014) differs from ours in that it did not reproduce the gait transition from tetrapod to tripod and did not exhibit adaptability against changes in the weight distribution and robustness against initial conditions. Furthermore, our approach is unique; we have discussed the common underlying mechanism of interlimb coordination in the locomotion of both vertebrates and arthropods by using legged robots.

The proposed interlimb coordination model shows adaptability to changes in the weight distribution of the robot's body, where the gait pattern did not change to a tripod gait but maintained a tetrapod gait after changing ω and the average duty factor of the loaded hind legs automatically became larger than those of the unloaded fore legs. These results were reproduced in a self-organizing manner by using Tegotae-based control, without any need to provide prior data about these changes. We additionally obtained biological evidence for the adaptability to changes in the weight distribution by conducting experiments using two crickets (*Gryllus bimaculatus*). These experiments are described in the Supplementary Material in detail. Our results using the robot clearly show good agreement with our biological evidence of the influence of the load on the leg coordination in crickets: with a load, (1) they exhibit a tetrapod gait and (2) increase the duty factor of the middle and hind legs. Furthermore, another experiment using fruit flies confirmed the same effect of a vertical load (Mendes et al., 2014), which suggests that such adaptability is observed for various species of insects. This fact strongly supports that the essentiality of using vertical GRFs $N_{i}^{V}$ for sensory information *S*(*N*) when designing a Tegotae function for hexapedal interlimb coordination.

Furthermore, our model exhibited adaptability to the physical conditions resulting from a two-leg amputation. If we use a predefined neural connection for a tripod gait—where the (L1, R2, L3) and (R1, L2, R3) legs are in-phase—, we cannot reproduce a trot or an L-S walk pattern—where the (L1, R3) and (R1, L3) feet alternately touch the ground in nearly anti-phase—when the two legs are amputated (Figure 11). Owing to the Tegotae-based interlimb coordination mechanism using both local (*N*_*i*_) and neighboring (*N*_*j*_) load information (Equation 9), gait patterns were self-organized in response to load distribution stemming from the remaining number of legs, which is one of the advantages of our approach. Some biological studies have suggested that insects generally exhibit the L-S walk when their two middle legs are amputated. Hughes (1957) have shown that two-middle-leg amputee cockroaches exhibited a gait—the touch-down order was (L3, L1, R3, R1), i.e., the L-S walk in quadrupeds. Graham (1977) and Grabowska et al. (2012) have shown that two-middle-leg amputee stick insects exhibited the same gait as cockroaches (Hughes, 1957) because the contralateral touch down timing became same such that gaits could be symmetric about the body axis to ensure its stability. Here, we did not actually conduct various leg-amputation tests; we can expect adaptability to some extent against some conditions, e.g., amputating a front/hind leg, owing to the potential of our model, as we have shown. However, because our model did not include any directional or posture controls and learning algorithms as in Ren et al. (2015) and Cully et al. (2015) (here, we mainly focus on real-time adaptability), its direction of motion would vary according to the physical properties: a front-left-leg amputated robot will turn left when moving forward. According to the patterns of leg amputation, insects exhibit modulation of their spatial footfall patterns, i.e., they change the landing location of a stance leg to maintain their posture stability (Hughes, 1957; Graham, 1977; Cruse, 1983; Grabowska et al., 2012); thus, we intend to apply an additional Tegotae-based controller for the modulation of spatial footfall patterns, resulting in the adaptation to a large number of leg amputations.

In insect locomotion, it is well known that two types of sensory signals play an essential role in leg coordination: (1) sensory signals about the position and velocity of joints during movement (Büschges, 2005; Pearson et al., 2006) and (2) force signals from the leg segments (Pearson, 1972; Bässler, 1977; Cruse, 1985a,b; Duysens et al., 2000; Zill et al., 2004). Such sensory signals modulate not only the timing (phase) but also the magnitude of neural output stems from the nervous system, e.g., CPGs (Grillner, 2003; Büschges, 2005). In our Tegotae-based approach, as a first step for the investigation, we use only vertical GRFs $N_{i}^{V}$ detected by force sensors installed in the legs to modulate the phase of oscillators. The obtained control principle, where both local and neighboring leg load information is essential for the interlimb coordination, agrees with biological evidence (Pearson, 1972; Bässler, 1977; Cruse, 1985a,b; Duysens et al., 2000; Zill et al., 2004). To reproduce increased adaptability to different surfaces and typed of movement, e.g., uneven terrain, uphill/downhill, similar to insects, other types of sensory signals, e.g., horizontal GRFs, would requires us to design additional Tegotae functions. Furthermore, modulation of the magnitude of motor output from neural systems will also lead to a change in landing location of a stance leg for negotiating various leg amputation patterns as discussed in the above paragraph. These topics seem to be of general interest and will also be studied in further investigations.

In the past two decades, various hexapod robots were developed with the aim of reproducing the adaptive functions of insects and to understand their control mechanisms (Kimura et al., 1993; Beer et al., 1997; Altendorfer et al., 2001; Ritzmann et al., 2004; Steingrube et al., 2010; Ambe et al., 2013; Manoonpong et al., 2013; Dasgupta et al., 2015; Ramdya et al., 2017). Ours was the first study of its kind to demonstrate various aspects of insect locomotion with a minimal control principle without any interlimb neural communication between oscillators. To the best of our knowledge, no studies have been reported in which adaptability was reproduced in a completely self-organized manner by only using local and neighboring load information. In the CPG approach as a control paradigm in this study, local sensory feedback *f*_*i*_ is described simply as a partial differential of the Tegotae function *T*_*i*_ with respect to the control variable ϕ_*i*_. This aspect of our model also suggests a new design scheme of local sensory feedback in the chain-of-reflex approach based on the discontinuous basic process, which should also be discussed as a next step. Our minimal model, which is systematically derived from the concept of Tegotae, is expected to provide substantial insight into the essence of the hexapedal interlimb coordination mechanism to roboticists as well as biologists.

## Author contributions

AI and DO conceived the research and managed the data collection. MG and SM designed the robot and conducted the experiments. MG, SM, and DO conducted the analyses. All authors wrote the manuscript together.

### Conflict of interest statement

The authors declare that the research was conducted in the absence of any commercial or financial relationships that could be construed as a potential conflict of interest.
