# Supplementary material for: A Minimal Model Describing Hexapedal Interlimb Coordination: The Tegotae-Based Approach
Source: Front Neurorobot. 2017 Jun 9;11:29. doi: 10.3389/fnbot.2017.00029 (PMC5465294; doi:10.3389/fnbot.2017.00029)
Supplement: Supplementary file 4 [file Presentation1.PDF]

frontiers

Supplementary Material: A Minimal Model Describing Hexapedal Interlimb Coordination: the Tegotae-based Approach

Dai Owaki [1], **Masashi Goda** [1], **Sakiko Miyazawa** [1], **and Akio Ishiguro** [1,2*]
*Correspondence:
Dai Owaki
owaki@riec.tohoku.ac.jp

1 BIOLOGICAL EXPERIMENT: ADAPTABILITY TO CHANGE IN WEIGHT DISTRIBUTION

We conducted experiments using two crickets (*Gryllus bimaculatus*, subject 1 with a body length of 2.3 cm and a body weight of 0.802 g and subject 2 with a body length of 2.5 cm and a body weight of 0.898 g) with a weight of 1.32 g on their backs. Figure S1 shows the experimental conditions in this biological experiment. We tested herein the effect of the weight on their backs on the gait patterns and the duty factor of each leg through five trials on two subjects ($N = 2, n = 5$). In the experiments, we observed a forward locomotion on a flat plane and recorded the top views using a high-speed video camera (i.e. DITECT, type HAS-L1) at a resolution of 640 $\times$ 480 pixels and 300 fps. We obtained the gait diagram by measuring the timing of touch-down and lift-off in each leg tip (pretarsus) by visually analyzing the recorded videos. Figure S2 shows the representative gait diagrams obtained in these experiments: (top graph) without load and (bottom graph) with load. These results indicated that crickets exhibited a tripod gait in the condition without load and a tetrapod gait in the condition with load. This fact corresponds to our experimental results in Fig. 9 and 10 ($\omega = 4.0$) in the main text.

Figure S3 compares the average duty factors of the front, middle, and hind legs between conditions without and with the load through five trials on two crickets. The result corresponds to our experimental results except for the duty factor in the fore legs. The lack of agreement in the duty factor in the front legs is owing to the limitation of the weight location according to the skeletal structure of crickets.

2 ROBOT EXPERIMENT: ASYMMETRIC WEIGHT DISTRIBUTION

We verified the effect of asymmetric load distribution on hind legs. Figure S4 shows the experimental result for asymmetric load distribution on hind legs, where we applied 270 g on left hind leg. This result indicated that our model can negotiate asymmetric weight distribution on their body by using local and neighboring load feedback mechanism.

3 QUANTITATIVE ANALYSIS OF ROBOT LOCOMOTION: AVERAGE DUTY FACTORS

The gait diagrams and movies of robot locomotion (i.e. movies S1 to S3) represent the qualitative performance of the robot locomotion, while the average duty factors obtained from the gait diagrams represent the quantitative performance as mentioned in the main text. We show herein three results concerning the quantitative performance of our robot with the use of the average duty factors:

1. Fig. S5: The duty factor decreased with the increasing ω, thereby indirectly indicating that the locomotion speed increased because of the decreasing feedback effects.
2. Fig. S6: The difference of the duty factors between the left and right legs indicated that the direction of the robot locomotion is not exactly straight because of a tiny asymmetricity of the robot structure.
3. Fig. S7: For the two-middle-leg amputation, increasing the local load in each leg resulted in the increasing duty factor for almost all legs, thereby leading to a decreasing locomotion speed.

We can also qualitatively confirm these results through movies S1 to S3.

4 STATE OF THE ART

Here, we summarize representative studies on the topics, (1) versatility of insect locomotion, and two main control paradigms on insect locomotion; (2) CPGs—neurophysiological findings and resulting models; and (3) chains of reflexes—behavioral findings and representative *Walknet* models.

4.1 Versatile insect locomotion

Previous biological studies have reported that insects exhibit versatile and adaptive locomotion according to locomotion speed, environmental condition, physical properties, and species, as shown in Table S1.

4.2 Control paradigms for insect locomotion

From a control perspective, past studies have intensively argued mainly from two distinct control paradigms: (1) CPGs (Table S2) and (2) chains of reflexes (Table S3). We summarize these paradigms by introducing representative studies. However, different viewpoints from various research groups could lead to ambiguous definitions for these control paradigms, resulting that each concept is not apparently delimited against the other one.

Table S1 Versatile insect locomotion

Paper	Subject	Summary
[1] Hughes, *J. Exp. Biol.* **34**, 1957.	cockroach (*Blatta Orientalis*)	The results indicated that cockroaches exhibit adaptive modification of step timing and footfall point according to leg amputation patterns.
[2] Graham, *J. Comp. Physiol.* **81**, 1972.	1st instar and adult stick insect (*Carausius Morosus*)	1st instar stick insects exhibit tetrapod and tripod gait according to speed, whereas adult stick insects exhibit only tetrapod gait.
[3] Cruse, *J. Comp. Physiol.* **112**, 1976.	stick insect (*Carausius Morosus*)	Walking on four different conditions: on a horizontal path, on a horizontal plane, on a horizontal beam, and up a vertical path. The results indicated the modification of leg trajectory and AEP/PEP(Anterior/Posterior Extreme Position).
[4] Graham, *J. Comp. Physiol.* **116**, 1977.	1st instar stick insect (*Carausius Morosus*)	The results compared three different conditions: intact, amputated, and leg restrained. Based on these results, the author proposed a control model that reproduces amputated and leg restrained patterns.
[5] Foth et al., *Biol. Cybern.* **47**, 1983.	adult stick insect	Load parallel to the body axis on treadwheel showed ipsilateral effect on AEP/PEP, which were modulated forward.
[6] Foth et al., *Biol. Cybern.* **48**, 1983.	adult stick insect	The results showed contralateral effect of load parallel to the body axis on treadwheel on AEP/PEP, which indicated strong coupling between contralateral leg for gait stability.
[7] Dean, *J. Exp. Biol.* **159**, 1991.	stick insect (*Carausius Morosus*)	They also investigated the inspilateral effect of load parallel to the body on treadwheel. The results indicated that the load affects on not only walking period but also footfall point.
[8] Zollikofer, *J. Exp. Biol.* **192**, 1994.	four types of ants (*Formicinae: Cataglyphis, Formica, Lasius; Myrmicinae: Myrmica*)	The results showed that ants exhibit tripod gait over wide range of locomotion speed. The stride length increased according to speed. These results were observed for four species of ants.
[9] Zollikofer, *J. Exp. Biol.* **192**, 1994.	four types of ants (*Formicinae: Cataglyphis, Formica, Lasius; Myrmicinae: Myrmica*)	The results showed that the stride length was a function of velocity, leg length, and additional parameter that depends on the species. Worker ants exhibit tripod gait, whereas female and male ants exhibit metachronal gait in *Cataglyphis Bombycina*.
[10] Zollikofer, *J. Exp. Biol.* **192**, 1994.	ant (*Cataglyphis fortis*)	The results indicated that carrying load did not affect gait patterns but affected footfall position and stride length.
[11] Noah et al., *J. Comp. Physiol.* **190**, 2004.	cockroach	The subjects that underwent leg amputation but had a prosthetic leg attached exhibited natural gait patterns, suggesting that feedback from receptors in proximal leg segments play an essential role in leg coordination.
[12] Goldman et al., *J. Exp. Biol.* **209**, 2006.	cockroach	The results showed the profile of ground reaction forces when cockroaches climbed up the tree.
[13] Sponberg et al., *J. Exp. Biol.* **211**, 2008.	cockroach	Cockroaches did not change gait patterns and duty factors on uneven terrain.
[14] Grabowska et al., *J. Exp. Biol.* **215**, 2012.	adult stick insects	Stick insects exhibit various adaptive gait patterns according to leg amputation patterns.
[15] Wosnitza et al., *J. Exp. Biol.* **216**, 2012.	drosophila	Dorsophia exhibits gait transition from tetrapod to tripod according to speed.

Table S2 Central Pattern Generators.

Paper	Subject	Summary
[17] Pearson et al., *J. Exp. Biol.* **58**, 1973.	cockroach (*Periplaneta american*)	The results showed the neurophysiological evidence, e.g. roles of motoneuron and sensory feedbacks, for interlimb coordination in cockroaches, and proposed a control model for a single leg based on these findings.
[18] Bässler et al., *J. Exp. Biol.* **105**, 1983.	stick insect (*Carausius morsus*)	The authors reported that the denerved thoracic ventral cord produced a motor output similar to that on stepping and rocking in intact.
[21] Bässler, *Biol. Cybern.* **54**, 1986.		Review—The definition of central pattern generators based on physiological studies.
[22] Bässler, *Biol. Cybern.* **69**, 1993.	stick insect (*Cuniculina impigra*)	This study revealed a modular system composed of reflex chains and endogenous oscillators for walking and searching in individual leg.
[23] Ryckebusch et al., *J Neurophysiol.* **69**, 1993.	locust	The results showed that rhythmic activity was induced in leg motor neurons when an isolated metathoracic ganglion was super-fused with the muscarinic agonist pilocarpine.
[24] Büschges et al., *J. Exp. Biol.* **198**, 1995.	stick insect	The deafferented stick insect thoracic nerve cord induced long-lasting rhythmic activity in leg motoneurons.
[25] Bässler and Büschges, *Brain Res Rev* **27**, 1998.	stick insect	Review—This article summarized the results obtained from neurophysiological investigations on the generation of leg movements during walking in a stick insect. .
[26] Büschges et al., *Eur J Neurosci.* **19**, 2004.	stick insect	The authors showed that alternating rhythmic motoneuron activity in the deafferented stick insect walking system resulted from phasic inhibitory drive provided by central pattern generating networks.
[27] Büschges, *J Neurosci.* **93**, 2005.	stick insect and cat	Review—The author reviewed common control schemes about central pattern generating networks with sensory feedback, controlling multi-segmented legs.
[28] Borgmann et al., *J Neurosci.* **29**, 2009.	stick insect (*Carausius morsus*)	The results showed that front-leg stepping in isolation resulted in in-phase activity of all ipsilateral legs, and functional stepping gaits emerge because of local load sensory feedback overriding the in-phase influence.
[29] Marder et al., *Curr. Biol.* **11**, 2011.	crayfish, locust, and stick insect	Review—Leg coordination mechanism based on CPGs as well as reflex mechanism in various arthropods.

Table S3 Chains of reflexes.

Paper	Subject	Summary
[31] Cruse, *Biol. Cybern.* **49**, 1983.	stick insect	The author modeled interlimb coordination based on various biological behavioral findings and reproduced gait patterns similar to insects' gait.
[32] Cruse, *Trends Neurosci.* **13**, 1990.	stick insect and crayfish	Review – The author summarized biological control mechanism from behavioral and neurophysiological evidences in stick insect and crayfish.
[33] Cruse et al., *Neural Netw.* **11**, 1998.	stick insect	The authors reviewed basic behavioral properties of hexapod walking and described a simple neural network called *Walknet*, which reproduced these properties.
[34] Dürr et al., *Arthropod Struct Dev.* **33**, 2004.	stick insect	The authors discussed the limitation of previous *Walknet* model and introduced its extension and the additionally reproduced abilities.
[35] Schilling et al., *Biol. Cybern.* **107**, 2013.	stick insect	The reviews compared the different *Walknet* versions to other approaches describing insect-inspired hexapod walking.

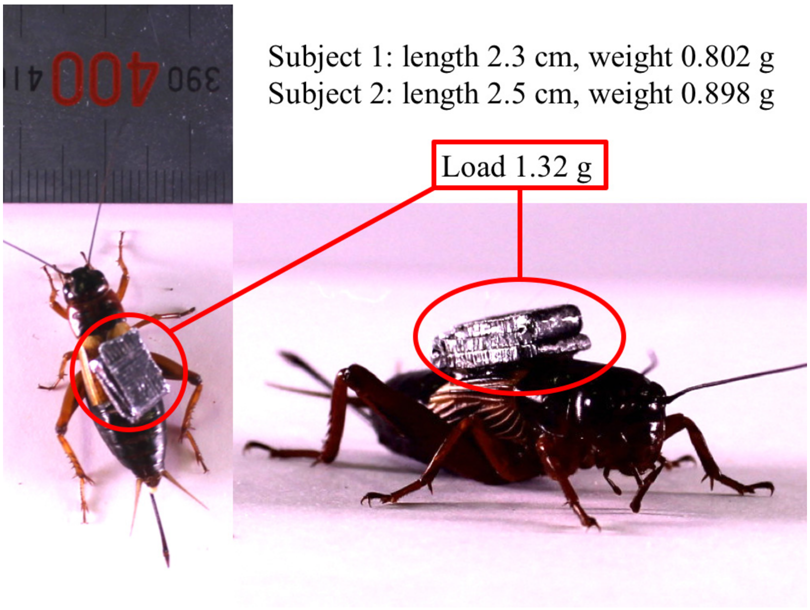

Figure S1. Left and right: top and oblique view of a subject: cricket (*Gryllus bimaculatus*).

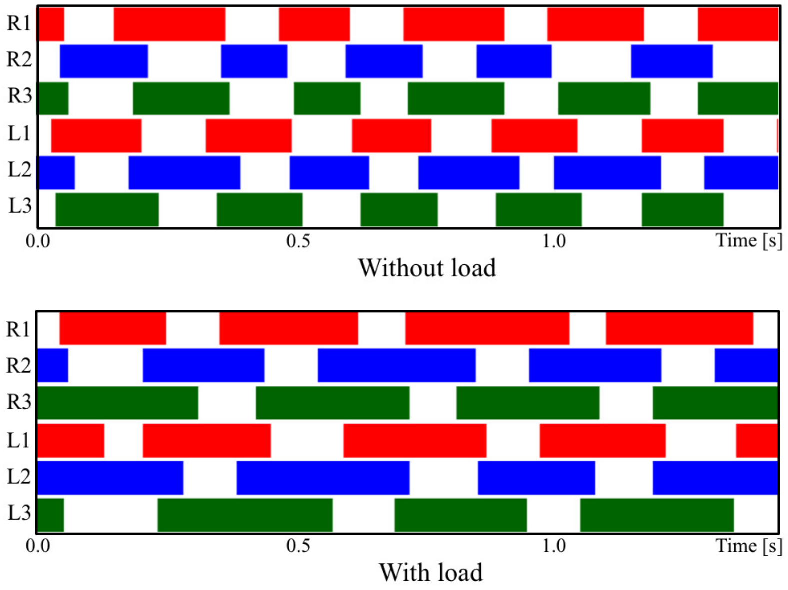

Figure S2. Representative gait patterns in the case without (top graph) and with (bottom graph) load

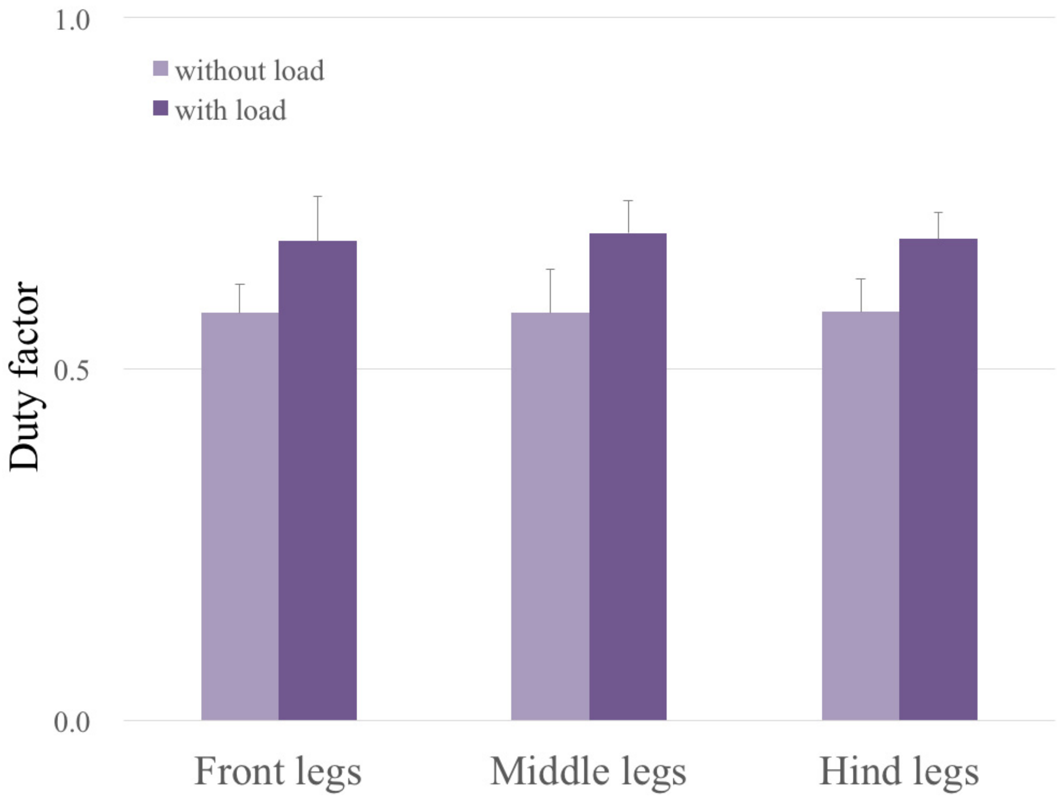

Figure S3. Average duty factor of each leg between conditions without and with the load through five trials on two crickets.

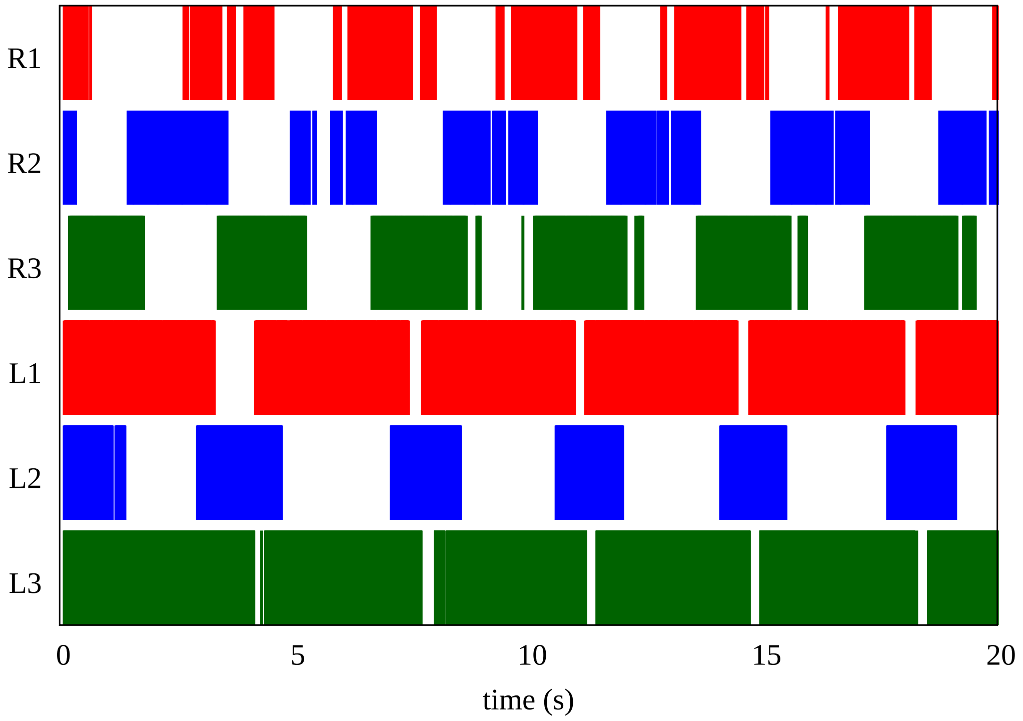

Figure S4. Condition of asymmetric load distribution on hind legs: 270 g on left hind leg.

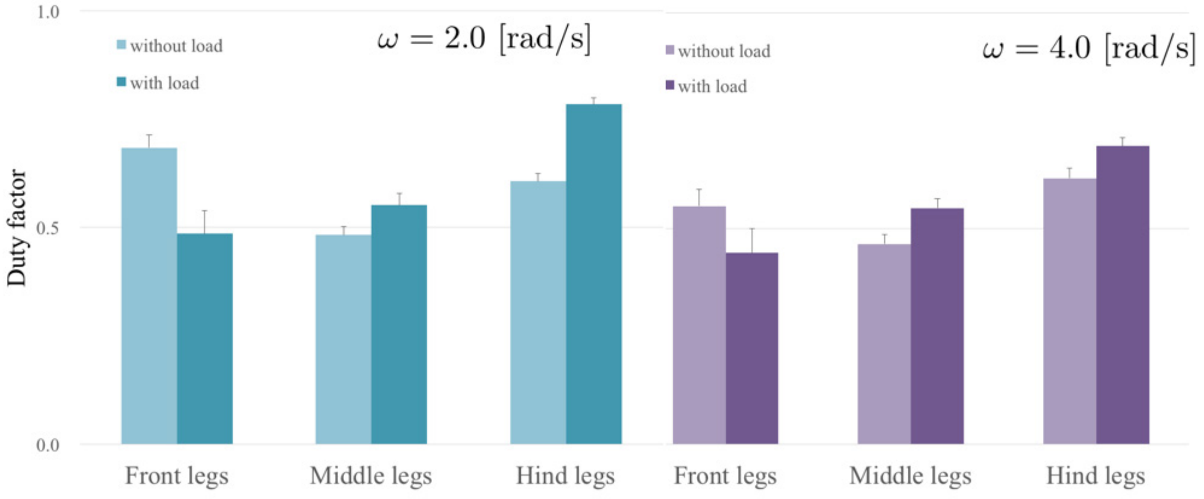

Figure S5. Duty factor decreased according to the increasing ω, thereby indirectly indicating that the locomotion speed increased (movies S1 and S2).

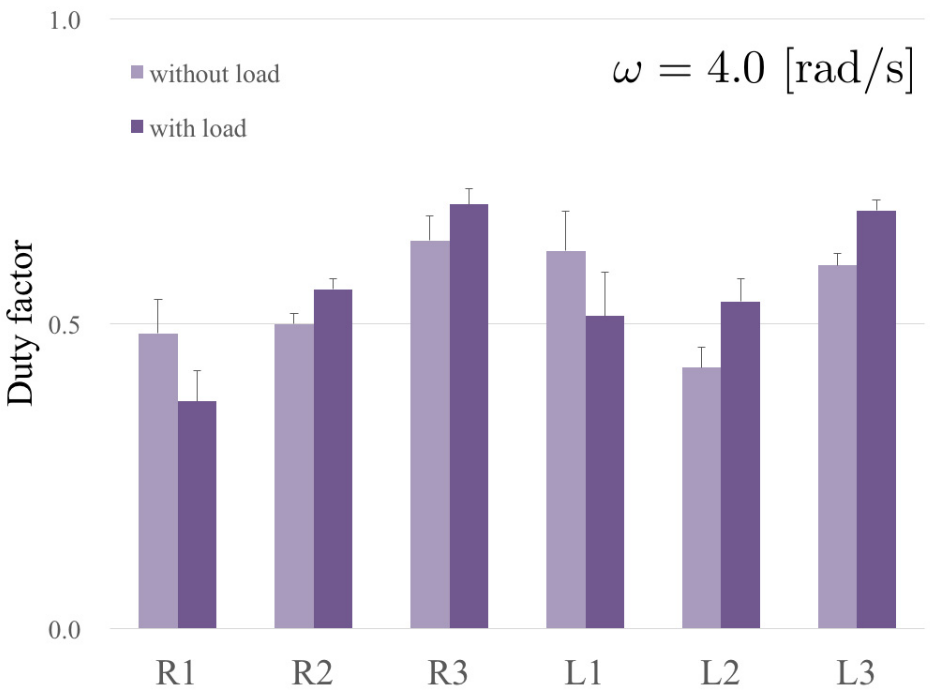

Figure S6. Asymmetric duty factors in the left and right legs indicating that the robot was (a bit) turning because of its tiny physical asymmetricity (movies S1 and S2).

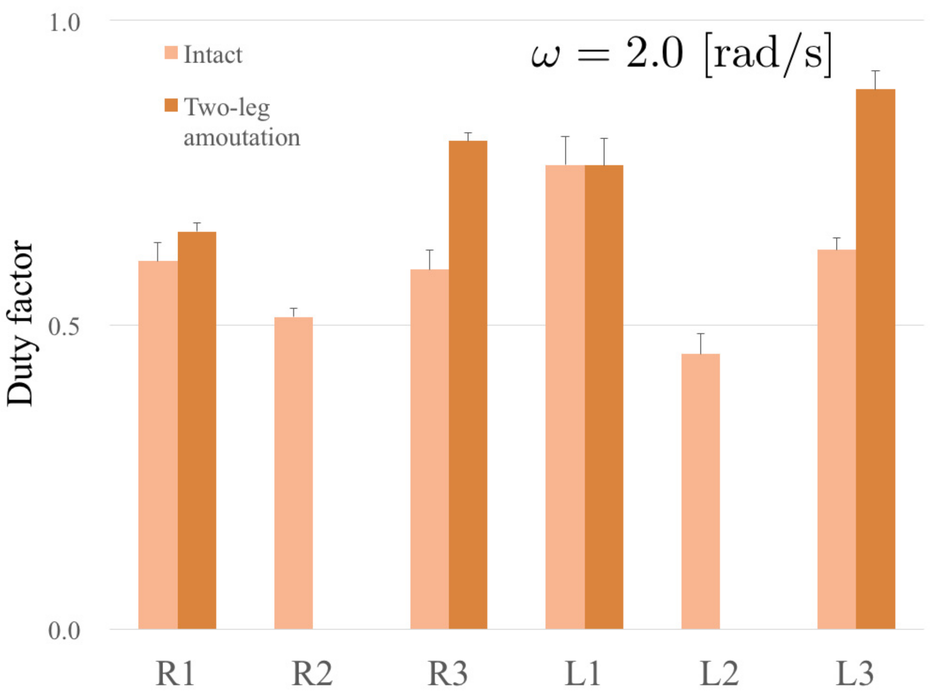

Figure S7. Duty factors increased in the case of the two-leg amputation. Increasing the local load in each leg owing to the physical property resulted in the increasing duty factors, thereby leading to a decreasing locomotion speed (movies S1 and S3).
